# Long Noncoding RNA LINC00202 Promotes Tumor Progression by Sponging miR-3619-5p in Retinoblastoma

**DOI:** 10.1247/csf.18033

**Published:** 2019-03-23

**Authors:** Guigang Yan, Yi Su, Zhao Ma, Lianzhi Yu, Ning Chen

**Affiliations:** 1 Department of Ophthalmology, the Affiliated Yantai Yuhuangding Hospital of Qingdao University, No 20 Yudong Road, Zhifu District, Yantai 264000, P.R. China; 2 Department of Radiation Oncology, the Affiliated Yantai Yuhuangding Hospital of Qingdao University, No 20 Yudong Road, Zhifu District, Yantai 264000, P.R. China; 3 Department of Physical Examination, the Affiliated Yantai Yuhuangding Hospital of Qingdao University, No 20 Yudong Road, Zhifu District, Yantai 264000, P.R. China

**Keywords:** LINC00202, miR-3619-5p, retinoblastoma, progression, RIN1

## Abstract

Retinoblastoma (RB) is the most common intraocular malignancy in childhood, and the prognosis in the advanced RB is poor. It is urgent to find novel therapeutic targets. Long noncoding RNAs (lncRNAs) have critical functions in cancer progression, and lncRNA LINC00202 is found associated with poor prognosis in RB. However, the functions of LINC00202 in RB remain unclear. We employed qRT-PCR and immunoblot to detect the expression levels of mRNAs and proteins, respectively. Cell proliferation was determined by CCK-8 assay and colony formation assay. Transwell assays were applied to evaluate the cell abilities of migration and invasion. Luciferase reporter assay was applied to examine RNA stability, and RNA pulldown assays were used to detect interaction between lncRNA and microRNA (miRNA). LINC00202 expression in RB tissues is higher than that in the paired adjacent normal tissues, which has correlation with poor prognosis in RB. RB cell proliferation, migration and invasion were weakened by LINC00202 depletion, but enhanced by LINC00202 overexpression. MiR-3619-5p was identified to directly bind and mediate LINC00202-promoted RB progression, meanwhile, miR-3619-5p directly regulated expression of an oncongene, RIN1. Moreover, RIN1 knockdown completely blocked miR-3619-5p-enhanced RB progression. In summary, high LINC00202 levels are correlated with poor prognosis in RB, and it promotes RB progression by sponging miR-3619-5p and therefore up-regulating RIN1 expression.

## Introduction

Retinoblastoma (RB) is the most common malignant neural retinal intraocular tumor of childhood, affecting one out of 16,000–18,000 neonates (Mansoor *et al.*, 2016; Villegas *et al.*, 2013). RB is curable at the early stage, and the survival rates of the patients with early stage RB are nearly 100% (Broaddus *et al.*, 2009). However, the survival rates are dramatically declined in undeveloped countries or regions, only 20–46% in Africa (Bowman *et al.*, 2008) due to delayed diagnosis and treatment (Dimaras *et al.*, 2010). The advanced RB can grow into the periocular tissues and finally into the brain, and even metastases to distant organs leading to death (Villegas *et al.*, 2013). Although some treatments have been developed to benefit RB patients, the prognosis in the advanced RB is still poor (Sengupta *et al.*, 2016). Thus, it is urgent to discover new effective therapeutic targets.

Long noncoding RNAs (lncRNAs) and microRNAs (miRNAs) are two classes of important RNA regulators engaged in cancer development (Ayers and Vandesompele, 2017). LncRNAs are a kind of RNAs more than 200 nucleotides in length that do not encode proteins (Quinn and Chang, 2016). LncRNAs regulate multiple biological processes, including cell proliferation, tumorigenesis and metastasis (Wang and Chang, 2011). For instance, lncRNA BANCR is involved in RB cell growth and metastasis, and its higher levels are related to poor prognosis in RB (Su *et al.*, 2015). RB development is regulated by lncRNA BDNF-AS (Shang *et al.*, 2018). MiRNAs are small RNAs with a length of 21–25 nucleotides and inhibit gene expression by either degrading the targeted transcripts or inhibiting translation of the targeted mRNAs. One approach of lncRNA exerting its functions is to sponge miRNAs and down-regulate their levels, that in turn up-regulates the expression levels of miRNA-targeted genes (Quinn and Chang, 2016). For example, lncRNA MALAT1 elevates Slug expression via miR-124 to modulate RB cell proliferation, migration and invasion (Liu *et al.*, 2017). H19 directly binds miR-17-92 to de-suppress p21 expression, and consequently prevents RB progression (Zhang *et al.*, 2018a). LINC00202 is a lncRNA related to poor prognosis in RB. It has been reported that it is also associated with overall survival (OS) time in patients with renal cancer (Liu *et al.*, 2018). However, the biological functions of LINC00202 have not been clarified.

The expression of Ras and Rab interactor 1 (RIN1) expression increases and is related to poor prognosis in many tumors, including non-small cell lung cancer (Wang *et al.*, 2012), melanoma (Fang *et al.*, 2012), gastric adenocarcinoma (Yu *et al.*, 2012) and renal cancer (Wei *et al.*, 2015). It has been reported that RIN1 overexpression aggravated tumor cells through activating EGFR signaling (Feng *et al.*, 2017). RIN1 is predicted to be one of the potential targets of miR-3619-5p, while miR-3619-5p was predicted to be one of LINC00202 targets. In this study, we investigated the functions of LINC00202 in RB and the underlying mechanism.

## Methods

### Tissue samples and Patients

Fifty cases of RB tissues and the paired adjacent normal tissues were obtained from the Affiliated Yantai Yuhuangding Hospital of Qingdao University. Once dissected out of patients, the tissues were immediately frozen in liquid nitrogen until use. This study had been approved by The Human Research Ethics Committee of the hospital and patients had written informed consents.

### Quantitative reverse transcription polymerase chain reaction (qRT-PCR)

To detect the levels of mRNA levels, total RNA was purified by TRIzol (Invitrogen, Carlsbad, CA). High Capacity cDNA Reverse Transcriptase kit (Applied Biosystems, Darmstadt, Germany) was applied to reverse transcribe 1 μg RNA to cDNA, and the expression levels were determined by quantitative PCR with SYBR Green Master Mix kit (ThermoFisher, Waltham, MA). The housekeeping gene, GAPDH, was used as the internal control. The following primers were used for qPCR, LINC00202, 5'-TCAGTGGGTGTCCTCATTGGT-3' and 5'- GCACAGTTTCATCCTCCTTCC-3'; RIN1, 5'-GCACCTGGCGAGAGAAAAG-3' and 5'-TAGATTTCCGCACGAGGAACG-3'; GAPDH, 5'-ACAACTTTGGTATCGTGGAAGG-3' and 5'-GCCATCACGCCACAGTTTC-3'. MiRNA was extracted with miRNeasy mini kit (Qiagen Hilden, Germany), and reversed transcribed with Omniscript reverse transcription kit (Qiagen) with the following primers, 5'-GTCGTATCCAGTGCAGGGTCCGAGGTATTCGCACTGGATACGACGCTGCA-3' for miR-3619-5p and 5'-GTCGTATCCAGTGCAGGGTCCGAGGTATTCGCACTGGATACGACAAAATATGGAA-3' for the internal control gene U6. Then qPCR was run to analyze the levels of miR-3619-5p and U6 with the following primers: miR-3619-5p, 5'-TCATCAGCAGGCAGGCTGGTGC-3' and 5'-GTGCAGGGTCCGAGGT-3'; U6, 5'-TGCGGGTGCTCGCTTCGGCAGC-3' and 5'-GTGCAGGGTCCGAGGT-3'.

### Cell culture and establishment of stable cell lines

The cells were cultured in RPMI-1640 (Gibco, ThermoFisher, Grand Island, NY, USA) containing 10% fetal bovine serum (FBS) (Hyclone, USA), and maintained in a humidified incubator at 37°C in the presence 5% CO_2_.

For establishment of cell lines stably expressing LINC00202 shRNAs, DNA fragments targeting LINC00202 sequences, 5'-GCTCTACCAAAGCGATCATTA-3' and 5'-GTGCTCTCAAGTCCGAAGTTT-3' were cloned into lentiviral vector PLKO. Y79 cells were transfected with LINC00202 shRNA plasmids and the control vector, followed by puromycin selection for 1 week to establish the stable cell lines.

To establish the cell line stable expressing LINC00202, LINC00202 DNA fragment was amplified by PCR with primers 5'-CTAGAATTCGTCAGCCATATGCCT-3' and 5'-CTAGGATCCTGAACGTTTAACTTTTA-3'. The DNA fragment was cloned into pBabeMNires vector. Weri-Rb1 cells were transfected with LINC00202 overexpression plasmid and control vector, followed by puromycin selection for 1 week to obtain stable cell lines.

### Cell proliferation assay

CCK-8 assay and colony formation assay were conducted to measure cell proliferation. To execute CCK-8 assay using a CCK-8 assay kit (CCK-8 assay kit, Dojindo, Japan), cells were plated into 96-well plate and cultured for 24 h, followed by starvation by removal of FBS for 12 h prior to adding 10 μl CCK-8 reagent. The absorbance at 450 nm was examined. Each group had 6 repeats, and the average was calculated. To do colony formation assay, cells were plated into 6-well culture plate at a density of 1000 per well and cultured for 2 weeks in a cell incubator. The cells were stained with 0.1% crystal violet (Beyotime, Shanghai, China) for 0.5 h after fixed with 4% paraformaldehyde for 0.5 h. The number of colonies was counted under a microscope.

### Transwell migration and invasion assays

For transwell migration assay, 1×10^5^ cells were plated into the upper chamber of the transwell chamber (Millipore, Billerica, MA, USA). To do transwell invasion assay, 1×10^5^ cells were plated to the matrigel-coated upper chamber (BD Biosciences, San Jose, CA, USA). For both assays, the upper chamber was added with FBS-free medium, and the lower chamber was supplied with 10% FBS-contained medium. After cultured for 24 h, the cells staying on the upper surface of membrane were swiped off, and the membrane was dyed with 0.5% crystal violet in 20% methanol. The cell abilities to migration and invasion were assessed by counting the number of cells through the membrane.

### Luciferase reporter assay

MiR-3619-5p binding LINC00202 wildtype (wt) and mutant (mt) sequences were obtained by PCR amplification with the following primers, wt, 5'-CTACTCGAGGTCAGCCATATGCCT-3' and 5'-CTAGCGGCCGCTGAACGTTTAACTTTTA-3'; mt, 5'-GGGTCGCCGTGAAAGCGAAGACGTTGGCAGGGA-3' and 5'-TCCCTGCCAACGTCTTCGCTTTCACGGCGACCC-3'. The wild-type (WT) or mutated (MT) RIN1 3'UTR DNA fragments containing miR-3619-5p binding site were acquired by PCR amplification with the following primers, WT, 5'-CTACTCGAGCTTGAAGTGGCCA-3' and 5'-CTAGCGGCCGCCACAGTCTGGGGGCCCC-3', MT, 5'-GCGGGAGACCCTGAGCGTACCCA-3' and 5'-TGGGTACGCTCAGGGTCTCCCGC-3'. These DNA fragments were inserted into psiCHECK2. the luciferase reporter plasmids combined with synthetic negative control (NC) or miR-3619-5p were delivered into cells in 96-well plate by transfection with Lipofectamine 2000 (Invitrogen), and cultured for 24 h. After the cells were lysed in 1×Passive Lysis Buffer, the Dual Luciferase Reporter Assay System (Promega, Madison, WI, USA) was applied to detect the activities of Renilla luciferase and firefly luciferase.

### Biotin RNA pull-down assay

This assay was conducted as previously reported (Zhang *et al.*, 2016). Biotin-labeled sense or antisense oligos of LINC00202 were incubated with Y79 cell lysate for 1 h. The complex was pull down by streptavidin-coupled agarose beads (Invitrogen). Sense probes included 5'-(biotin-)AACCAGGATGTGGTCTGGTTGGCAGGGCAA-3', 5'-(biotin-)CAGGAGACGCCCAAAGGTAGGGAGGTGACA-3' and 5'-(biotin-)CGAAGTGGAGGTGGTAGTAGCGCAGGACCC-3'. Antisense probes comprised 5'-(biotin-)TTGCCCTGCCAACCAGACCACATCCTGGTT-3', 5'-(biotin-)TGTCACCTCCCTACCTTTGGGCGTCTCCTG-3' and 5'-(biotin-)GGGTCCTGCGCTACTACCACCTCCACTTCG-3'. The isolated RNA was transcribed into cDNA and then the amounts of LINC00202 and miR-3619-5p were measured by qPCR as described in the method of qRT-PCR.

### Western blot

Cells were lyzed in RIPA buffer (50 mM Tris-HCl, pH 7.5, 0.1% SDS, 1% Nonidet P-40, 0.1% sodium deoxycholate, 150 mM NaCl). The protein samples were subject to electrophoresis in SDS-PAGE gels, and then the separated proteins were transferred onto PVDF membranes (Millipore, Bedford, USA). The membranes were blocked with 5% non-fat milk, before incubated with primary antibodies to RIN1 (Abcam, Cambridge, MA, USA) and β-actin (Sigma, St. Louis, MO, USA) at 4°C overnight. After washed 3 times with washing buffer, the membranes were blotted with HRP-coupled secondary antibodies (Promega). The enhanced chemiluminescence Detection System (ThermoFisher, USA) was used to detect Protein bands.

### Statistical analysis

In this study, the data were expressed as the mean value±SD (standard deviation). *P* values were calculated by Student’s *t*-test or ANOVA analysis and *P*<0.05 was defined as statistical significance difference.

## Results

### LINC00202 is up-regulated in RB and high LINC00202 expression predicts poor prognosis

**Fig. 1:**
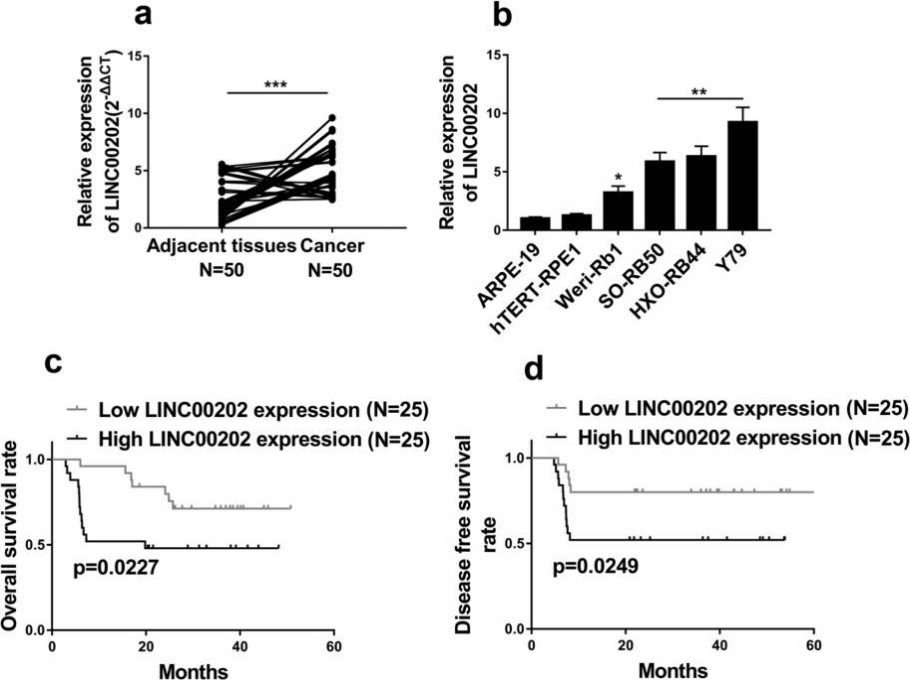
LINC00202 is up-regulated in RB and high LINC00202level predicts poor prognosis. (a) LINC00202 expression in RB tissues and the paired adjacent tissues was detected by qRT-PCR. (b) LINC00202 expression in RB cell lines (SORB50, Weri-Rb1, HXO-RB44 and Y79) and normal retinal pigment epithelial cell line (hTERT-RPE1, ARPE-19) was analyzed by qRT-PCR. (c and d) Kaplan–Meier survival curves show that high LINC00202 levels were associated with poor overall survival (OS) rate as well as disease-free survival (DFS) rate. The data display the mean±SD based on three independent experiments. *P<0.05; **P<0.01; ***P<0.001.

LINC00202 is one of the lncRNAs associated with overall survival (OS) time in patients with renal cancer (Liu *et al.*, 2018). To investigate if LINC00202 functions in RB, we first examined this lncRNA expression in RB tissues by qRT-PCR. LNC00202 expression in RB tissues was significantly richer comparing to the adjacent normal tissues (Fig. 1a). Similarly, LINC00202 mRNA levels expressed in RB cell lines, including Weri-Rb1, SORB50, HXO-RB44 and Y79, were 3 to 10 folds higher than those in normal retinal pigment epithelial cell lines, ARPE-19 and hTERT-RPE1 (Fig. 1b). These data demonstrate that LINC00202 is up-regulated in RB. Next, we explored correlation between LINC00202 expression levels and OS rate or disease-free survival (DFS) rate in 50 RB cases. We divided RB patients into 2 groups according to LINC00202 expression levels, i.e. low LINC00202 expression and higher LINC00202 expression, and each group contained 25 cases. Kaplan–Meier survival analysis revealed that the group of high LINC00202 expression had a significantly lower OS rate (Fig. 1c) and a lower DFS rate (Fig. 1d), suggesting that high LINC00202 expression level is related to poor prognosis and LINC00202 is an oncogene in RB.

### LINC00202 regulates RB cell proliferation, migration and invasion

**Fig. 2:**
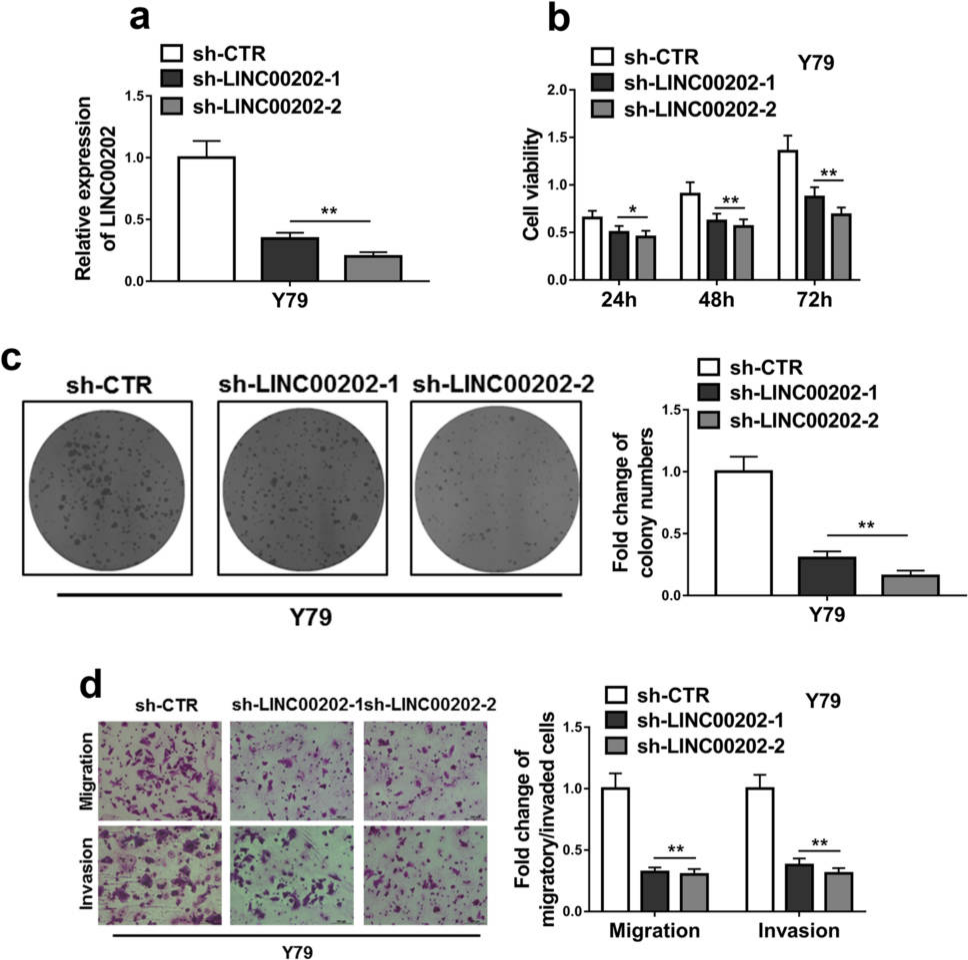
LINC00202 knockdown inhibits RB cell proliferation, migration and invasion. Y79 cells were transfected with LINC00202 shRNAs (sh-LINC00202-1, sh-LINC00202-2) or empty vector (sh-CTR), followed by puromycin selection to establish the stable cell lines. The stable cells were then employed for qRT-PCR analysis of LINC00202 expression (a), CCK-8 assay (b) and colony formation assay (c) to analyze cell viability, and transwell assays to examine cell abilities to migration and invasion (d). The data display the mean±SD based on three independent experiments. *P<0.05; **P<0.01.

To explore the function of LINC00202 in RB, LINC00202 expression in RB cells was disturbed by knockdown or overexpression. Y79 cells express the highest LINC00202 level in all examined RB cell lines, so we knocked down LINC00202 in Y79 cells. Two LINC00202 shRNAs both efficiently reduced LINC00202 expression, and the knockdown efficiency was over 60% (Fig. 2a). LINC00202 knockdown significantly retarded cell proliferation (Fig. 2b). Y79 ability of colony formation was also severely impaired by LINC00202 shRNAs (Fig. 2c). As shown in the right panel of Fig. 2c, the numbers of colonies in LINC00202 knockdown cells were reduced by over 60%. These data indicate that LINC00202 regulates Y79 cell proliferation. Meanwhile, transwell assays showed that Y79 abilities of both migration and invasion were declined by over 50% in response to LINC00202 knockdown (Fig. 2d). In contrast, LINC00202 overexpression in Weri-Rb1 cells (Fig. S1a), which expressed the lowest LINC00202 level among all examined RB cell lines (Fig. S1b), significantly promoted cell viability (Fig. S1b) and increased the number of colonies over 3 folds in the colony formation assay (Fig. S1c). Meantime, transwell assays unveiled that Weri-Rb1 abilities of both migration and invasion were elevated over 3 folds in response to LINC00202 overexpression (Fig. S1d).

In summary, LINC00202 is engaged in manipulating RB cell proliferation, migration and invasion.

### LINC00202 negatively modulates miR-3619-5p through direct interaction

**Fig. 3:**
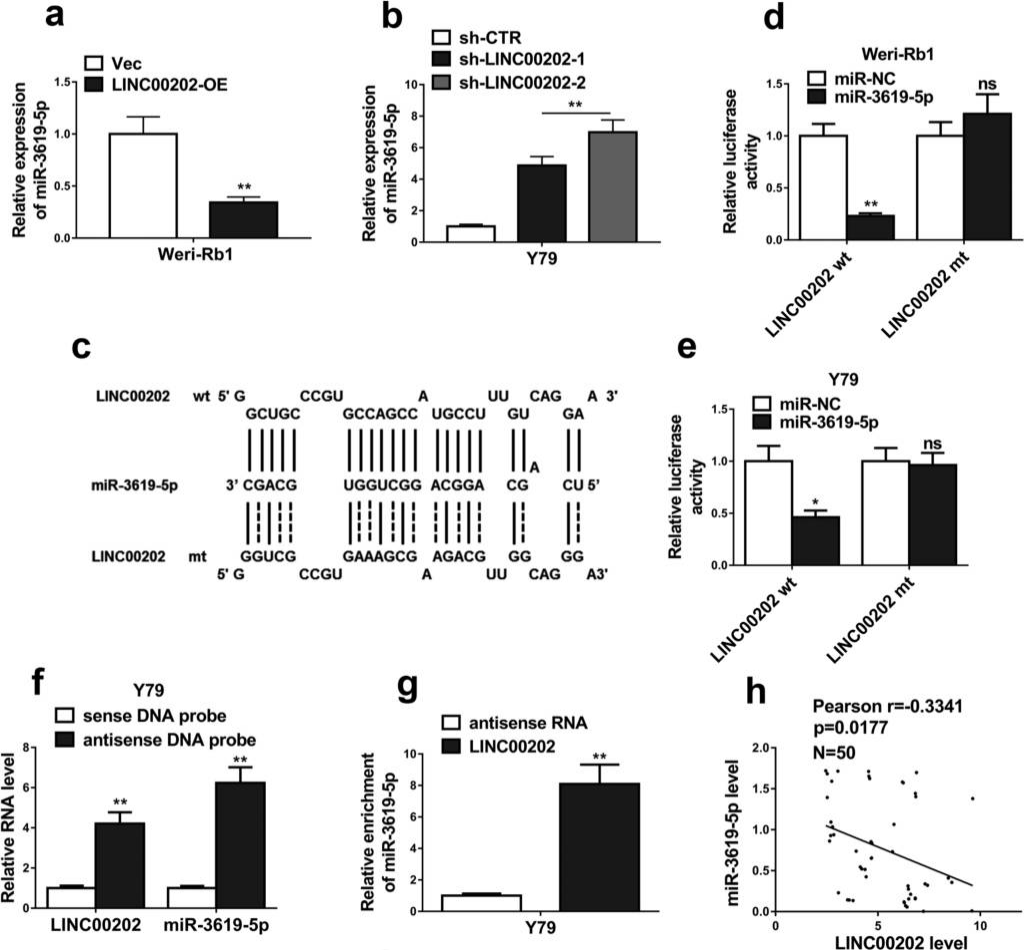
LINC00202 negatively modulates miR-3619-5p through direct interaction. qRT-PCR was performed to determinemiR-3619-5p expression in Weri-Rb1 cells stably overexpressing LINC00202 (a) and in Y79 cells stably expressing LINC00202 shRNAs (b). (c) miR-3619-5p binding site on LINC00202 transcript was predicted and the wild-type (wt) or mutated (mt) sequence ofmiR-3619-5p binding site on LINC00202 was cloned into a luciferase reporter vector, which were then co-transfected with miR-3619-5p or miR-NC mimics into Weri-Rb1 cells (d) and Y79 cells (e), and cultured for 24 h before the luciferase activities were determined. (f) Biotin-coupled sense or antisense DNA probes targetingLINC00202 were incubated with Y79 cell lysate to pull down RNAs, followed by qRT–PCR analysis of the amounts of LINC00202 and miR-3619-5p. (g) Biotin-labeled LINC00202RNA and antisense RNA were incubated with Y79 cell lysate to pull down RNAs, and subsequently qRT–PCR was performed to analyze the miR-3619-5p amount. (h) shows correlation between LINC00202 and miR-3619-5p expression levels in 50 RB tissue samples. The data in (a), (b) and (d–g) display the mean±SD based on three independent experiments. *P<0.05; **P<0.01.

To investigate the mechanism underlying how LINC00202 regulates RB cell progression, we predicated LINC00202 binding miRNAs, as lncRNA sponge of miRNAs is a general way to exert lncRNA functions (Quinn and Chang, 2016). We predicted that miR-3619-5p was one of LINC00202 targets by software miRcode (http://www.mircode.org/mircode/). LINC00202 overexpression greatly dampened miR-3619-5p expression (Fig. 3a), while LINC00202 knockdown elevated miR-3619-5p levels over 4 folds (Fig. 3b). These data indicated that miR-3619-5p is targeted by LINC00202. To elucidate if LINC00202 directly manipulates miR-3619-5p expression, a potential miR-3619-5p binding site on LINC00202 sequence was identified by software RNAhybrid (https://bibiserv.cebitec.uni-bielefeld.de/rnahybrid) (Fig. 3c). The potential miR-3619-5p-bound LINC00202 wild-type (wt) sequence or its mutated (mt) sequence (Fig. 3c) was cloned into a luciferase reporter vector. MiR-3619-5p greatly inhibited the activities of the luciferase fused with miR-3619-5p-bound wt LINC00202 sequence but not mt LINC00202 sequence in Weri-Rb1 cells (Fig. 3d) and Y79 cells (Fig. 3e). Furthermore, LINC00202 antisense probe pull down not only LINC00202 RNA but also miR-3619-5p (Fig. 3f). In addition, full-length LIN00202 RNA was able to enrich miR-3619-5p from Y79 cell lysate (Fig. 3g). By virtue of these data, we concluded that LIN00202 directly binds miR-3619-5p to regulate its expression. In 50 cases of RB tumors, the expression levels of LINC00202 were negatively correlated with miR-3619-5p expression levels (Fig. 3h), further supporting the above conclusion.

### MiR-3619-5p inhibits RB cell proliferation, migration and invasion

**Fig. 4:**
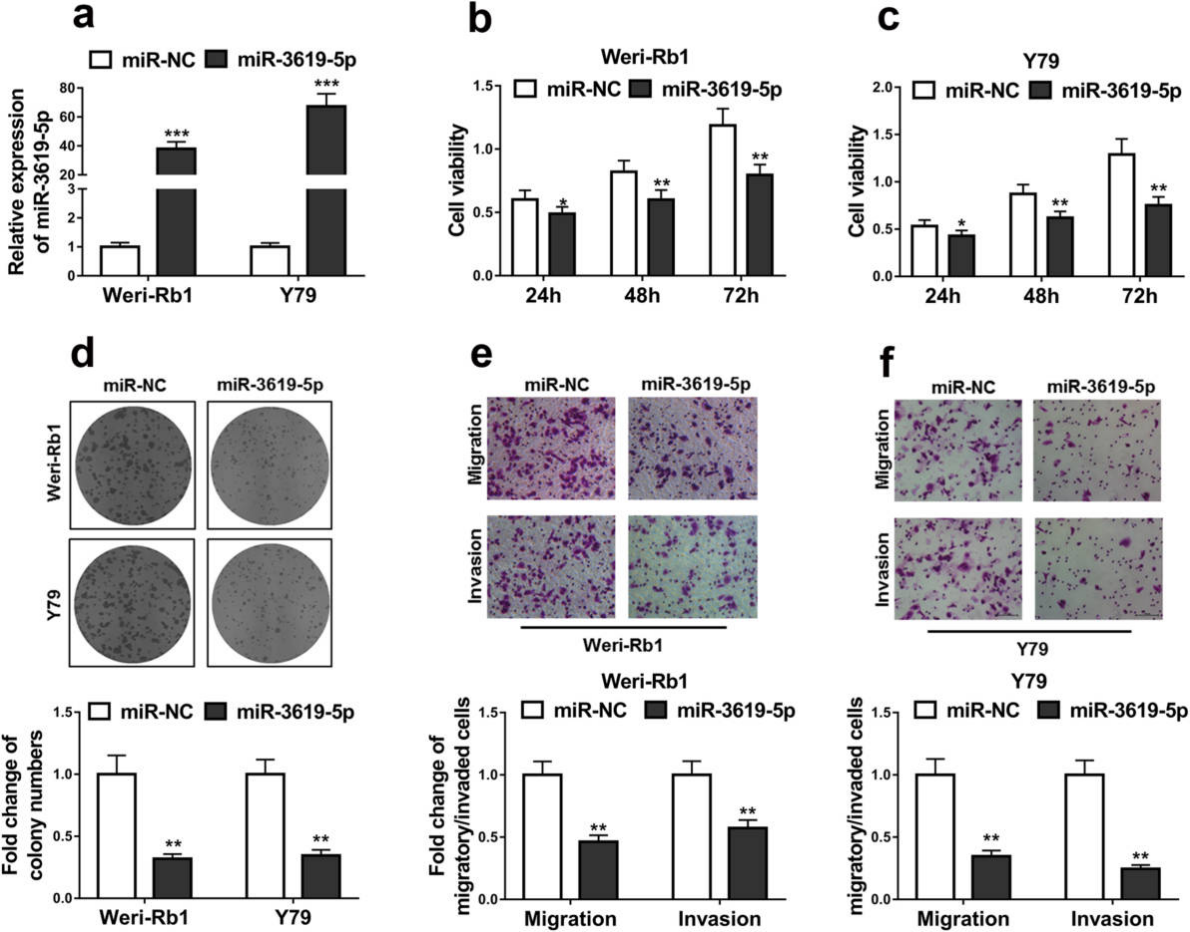
MiR-3619-5p inhibits RB cell proliferation, migration and invasion. Y79 and Wei-Rb1 cells were treated with negative control mimic (miR-NC) or miR-3619-5p mimic (miR-3619-5p). The cells were then used for qRT-PCR analysis of miR-3619-5p expression (a), CCK-8 assay (b, c) and colony formation assay (d) to analyze cell viability, and transwell assays to examine cell abilities to migration and invasion (e, f). The data display the mean±SD based on three independent experiments. *P<0.05; **P<0.01; ***P<0.001.

As there is direct interaction between miR-3619-5p and LINC00202, we asked whether miR-3619-5p had effects on RB cell progression. To address this speculation, synthetic miR-3619-5p or negative control miRNA were transfected into Weri-Rb1 and Y79 RB cells. MiR-3619-5p was efficiently transfected into the cells, as their levels were elevated over 40 folds (Fig. 4a). MiR-3619-5p overexpression significantly reduced cell viabilities of both Weri-Rb1 (Fig. 4b) and Y79 cells (Fig. 4c). In colony formation assay, the numbers of colonies were decreased by over 60% in both Weri-Rb1 and Y79 cells (Fig. 4d). Moreover, miR-3619-5p lowered Weri-Rb cell abilities of migration and invasion by 50% and that of Y79’s over 60% (Fig. 4e and 4f). These results demonstrated that miR-3619-5p has effects on RB cell proliferation, migration and invasion.

### LINC00202 modulates RB cell progression via regulating miR-3619-5p

**Fig. 5:**
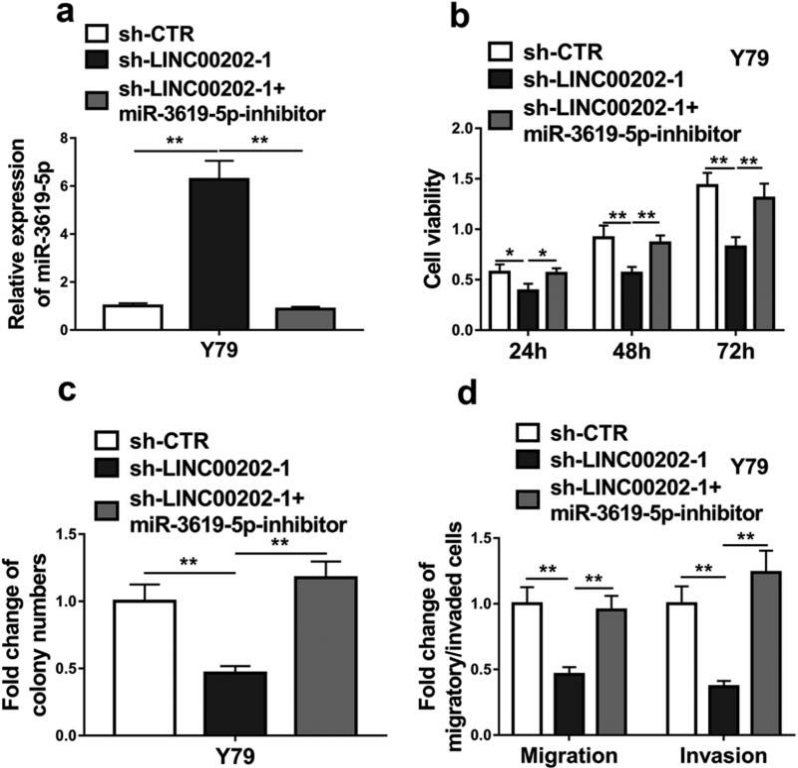
LINC00202 modulates RB cell progression via regulating miR-3619-5p. Y79 cells stably expressing LINC00202 shRNAs were transfected with negative control miRNA mimic (miR-NC) or miR-3619-5p mimic (miR-3619-5p). Then the cells were applied for qRT-PCR analysis of miR-3619-5p expression (a), CCK-8 assay (b) and colony formation assay (c) to analyze cell viability, and transwell assays to examine cell abilities to migration and invasion (d). The data display the mean±SD based on three independent experiments. *P<0.05; **P<0.01.

To investigate if LINC00202 and miR-3619-5p are in the same axis to regulate RB cell progression, miR-3619-5p inhibitor, the antisense oligonucleotide of the matured miR-3619-5p, was transfected into the cells stably expressing LINC00202 shRNA. MiR-3619-5p inhibitor completely blocked miR-3619-5p induction by LINC00202 shRNA (Fig. 5a), and totally eliminated the inhibition effect of LINC00202 knockdown on cell viability (Fig. 5b), the cell abilities of colony formation (Fig. 5c), migration and invasion (Fig. 5d). These results suggest that LINC00202 modulates RB cell progression via regulating miR-3619-5p.

### miR-3619-5p targets RIN1 to regulate RB progression

**Fig. 6:**
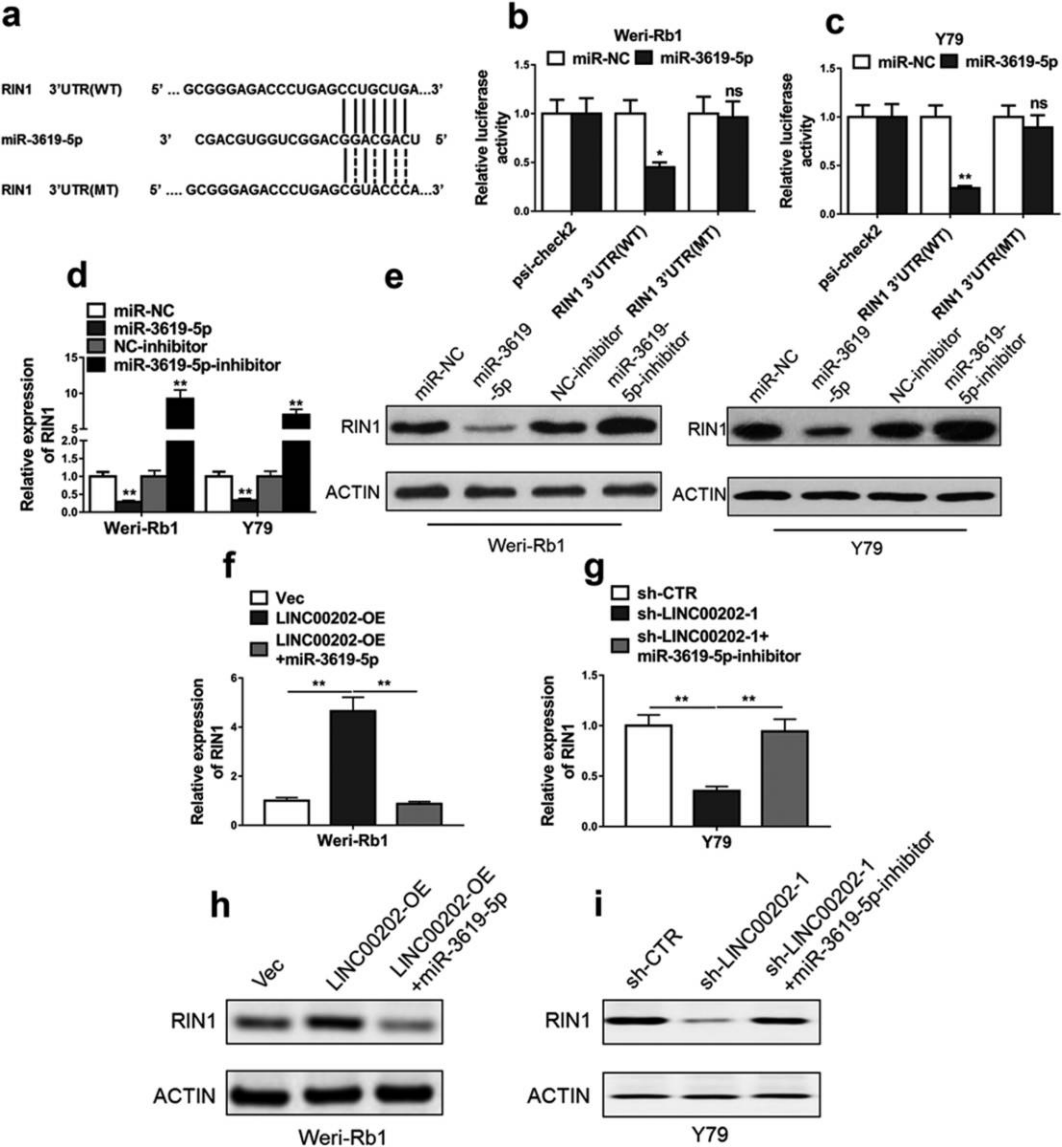
RIN1 is a direct target of miR-3619-5p in RB cells. MiR-3619-5p binding site within RIN1 3'UTR was predicted (a), and the DNA fragments of wild-type (WT) and mutant (MT) sequences (a) were cloned into luciferase reporter vector. Luciferase reporter assay was conducted in Weri-Rb1 (b) and Y79 cells (c) after co-transfection with these luciferase reporter plasmids and miR-3619-5p or miR-NC. Weri-Rb1 and Y79 cells were treated with miRNA mimic, miR-3619-5p or its inhibitor, and subsequently qRT-PCR and Western blot were conducted to detectbRIN1 mRNA levels (d) and protein levels (e), respectively. Weri-Rb1 cells stably overexpressing LINC00202 were treated with miR-3619-5p, or Y79 cells stably expressing LINC00202 shRNA were treated with miR-3619-5p inhibitor, and then qRT-PCR and Western blot were conducted to detect RIN1 mRNA levels (f, g) and protein levels (h, i), respectively. The data display the mean±SD based on three independent experiments. *P<0.05; **P<0.01.

We predicted that RIN1 was one of the potential targets of miR-3619-5p using software Targetscan (http://www.targetscan.org/vert_71/). RIN1 is associated with renal cell carcinoma aggressiveness (Feng *et al.*, 2017). The potential miR-3619-5p binding site on RIN1 gene was located in the 3' untranslated region (3'UTR) (Fig. 6a). The wild-type (WT) miR-3619-5p binding site sequence or the mutated (MT) one was cloned into a luciferase reporter vector, and were delivered into Rb cells combined with miR-3619-5p or control miRNA. The activities of the luciferase fused with WT RIN1 3'UTR, but not MT RIN1 3'UTR, were dramatically reduced by miR-3619-5p in both Weri-Rb1 cells (Fig. 6b) and Y79 cells (Fig. 6c). Furthermore, the expression levels of both endogenous RIN1 mRNA and protein levels were down-regulated by miR-3619-5p. Whereas up-regulated by miR-3619-5p inhibitor, in Weri-Rb1 and Y79 cells (Fig. 6d–e). These results implied that RIN1 is a direct target of miR-3619-5p. LINC00202 overexpression induced RIN1 expression in Weri-Rb1 cells, and this induction was abrogated by miR-3619-59 (Fig. 6f and 6h). In contrast, LINC00202 knockdown down-regulated RIN1 expression, that was rescued by miR-3619-5p inhibitor (Fig. 6g and 6i). All together, we conclude that RIN1 is directly regulated by miR-3619-5p in RB cells.

To address the importance of RIN1 in RB progression, RIN1 was overexpressed in Weri-Rb1 and Y79 cells, confirmed by qRT-PCR (Fig. S2a) and Western blot (Fig. S2b). RIN1 overexpression increased both cell lines’ cell viabilities (Fig. S2c and S2d), and the cells abilities of colony formation (Fig. S2e), cell migration and invasion (Fig. S2f and S2g). In Y79 cells, the RIN1 up-regulation by miR-3619-5p inhibitor was repressed by RIN1 siRNA (Fig. S2k and S2i). Consequently, miR-3619-5p-enhanced cell progression was completely blocked by RIN1 siRNA (Fig. S2j–l). These results suggest that RIN1 mediates miR-3619-5p-regulated RB progression.

## Discussion

This study showed that lncRNA LNC00202 was a biomarker for prognosis in RB, and higher LNC00202 expression could predict poor prognosis. Furthermore, we elucidated LNC00202 functions in RB cells and the related mechanism. LNC00202 promoted RB cell progression by inhibiting miR-3619-5p and thereby leading to up-regulation of miR-3619-5p targeted oncogene, RIN1. Thus, LNC00202, miR-3619-5p and RIN1 form an axis to modulate RB cell progression. This is the first study reporting LNC00202 pro-malignant functions as well as the LNC00202/miR-3619-5p/RIN1 regulation axis in RB.

Recently, lncRNAs have gained attention as their involvement in many physiological and pathological processes (Quinn and Chang, 2016). Accumulating literatures have highlighted that the expression change of lncRNAs has critical and clinically predictive roles in tumorigenesis (Elchuri *et al.*, 2018). More and more lncRNAs were reported to regulate RB development and progression, including XIST (Hu *et al.*, 2018), MT1JP (Bi *et al.*, 2018), LIN00152 (Li *et al.*, 2018), CCAT1 (Zhang *et al.*, 2017), BANCR (Su *et al.*, 2015), H19 (Zhang *et al.*, 2018a), BDNF-AS (Shang *et al.*, 2018), MALAT1 (Liu *et al.*, 2017) and HOTAIR (Dong *et al.*, 2016). Except for MT1JP and BDNF-AS, which are down-regulated and act as a tumor suppressor gene in RB, other lncRNAs are all up-regulated in RB and promote RB progression. This study found a new lncRNA in RB, LNC00202, functioning as an oncogene and promoting RB progression via inhibiting miR-3619-5p.

There are several evidences to demonstrate LNC00202 promoting RB progression via miR-3619-5p. First, the activity of the luciferase fused with the miR-3619-5p-bound wild-type LNC00202 sequence rather than the mutated sequence was inhibited by miR-3619-5p (Fig. 3d–e). Second, miR-3619-5p was pull down by LINC00202 antisense probes (Fig. 3f–g), indicating direct interaction between LNC00202 and miR-3619-5p. Third, LNC00202 depletion increased miR-3619-5p expression levels (Fig. 3b), while LNC00202 knockdown promoted RB progression (Fig. 2), phenocopied by miR-3619-5p overexpression (Fig. 4). These results suggested that LNC00202 and miR-3619-5p functions in the same axis. Fourth, LNC00202 knockdown-caused RB progression was completely abolished by miR-3619-5p inhibitor (Fig. 5). All together, we conclude that LNC00202 promotes RB progression through inhibiting miR-3619-5p.

Several studies have reported that miR-3619-5p acts as a tumor suppressor in various cancers. MiR-3619-5p decreases β-catenin expression to suppress progression of non-small cell lung cancer (Niu *et al.*, 2015) and bladder cancer (Zhang *et al.*, 2018b). MiR-3619-5p binds to the promoter of CDKN1A and induces its expression to inhibit prostate cell proliferation (Li *et al.*, 2017). This study shows that miR-3619-5p also had a tumor suppressive function in RB, and RIN1 was identified as a new miR-3619-5p target.

RIN1 possesses an oncogenic role via Ras signaling pathway (Han *et al.*, 1997). High RIN1 expression is related to tumor progression and poor prognosis in bladder urothelial carcinoma (Shan *et al.*, 2012), gastric adenocarcinoma (Yu *et al.*, 2012) , melanoma (Fang *et al.*, 2012) and non-small cell lung cancer (Wang *et al.*, 2012). Here we found that RIN1 expression was regulated by miR-3619-5p (Fig. 6), and is an effector to mediate miR-3619-5p anti-tumor function.

In conclusion, LINC00202 could be a prognosis biomarker for RB, and has an important role in RB cell proliferation, migration and invasion via inhibiting miR-3619-5p and consequently inducing RIN1 expression. Our results imply that LINC00202 could be a potential therapeutic target for RB, and miR-3619-5p is a promising drug candidate for RB therapy.

## Conclusion

High LNC00202 level is related to poor prognosis in RB, and LNC00202 promotes RB proliferation, migration and invasion via miR-3619-5p and its target RIN1.

## Acknowledgments

None.

## Funding

None.

## Conflict of interest

None.

## Supplementary Material

Fig. S1 LINC00202 overexpression promotes RB cell proliferation, migration and invasion. Weri-Rb1 cells were transfected with LINC00202 overexpression plasmid (LINC00202-OE) or empty vector (Vec), followed by puromycin selection to establish the stable cell lines. The stable cells were then subjected to qRT-PCR analysis of LINC00202 expression (a), CCK-8 assay (b) and colony formation assay (c) to analyze cell viability, and transwell assays to examine cell abilities to migration and invasion (d). The data represent the mean±SD from three independent experiments. ***P*<0.01; ****P*<0.001.

Fig. S2 RIN1 is a crucial effector for RB progression. Weri-Rb1 and Y79 cells were transfected with RIN1 overexpression plasmid (RIN1-OE) or empty vector (Vec), and then subject to qRT-PCR analysis of RIN1 mRNA levels (a), Western blot analysis of RIN1 protein levels (b), CCK-8 assay (c, d) and colony formation assay (e) to analyze cell viability, and transwell assays to examine cell abilities to migration and invasion (f, g). Y79 cells were transfected with miR-3619-5p inhibitor combined with RIN1 siRNA, followed by qRT-PCR analysis of RIN1 mRNA levels (h), Western blot analysis of RIN1 protein levels (i), CCK-8 assay (j) and colony formation assay (k) to analyze cell viability, and transwell assays to examine cell abilities to migration and invasion (l). The data represent the mean±SD from three independent experiments. The data represent the mean±SD from three independent experiments. **P*<0.05; ***P*<0.01; ****P*<0.001.
