# Supplementary material for: Long Noncoding RNA LINC00202 Promotes Tumor Progression by Sponging miR-3619-5p in Retinoblastoma
Source: Cell Struct Funct. 2019 Mar 23;44(1):51–60. doi: 10.1247/csf.18033 (PMC11926405; doi:10.1247/csf.18033)
Supplement: Supplementary file 1 — Fig. S1 LINC00202 overexpression promotes RB cell proliferation, migration and invasion. Weri-Rb1 cells were transfected with LINC00202 overexpression plasmid (LINC00202-OE) or empty vector (Vec), followed by puromycin selection to establish the stable cell lines. The stable cells were then subjected to qRT-PCR analysis of LINC00202 expression (a), CCK-8 assay (b) and colony formation assay (c) to analyze cell viability, and transwell assays to examine cell abilities to migration and invasion (d). The data represent the mean±SD from three independent experiments. **P<0.01; ***P<0.001. [file csf_44_18033_1.pdf]

Supplementary materials

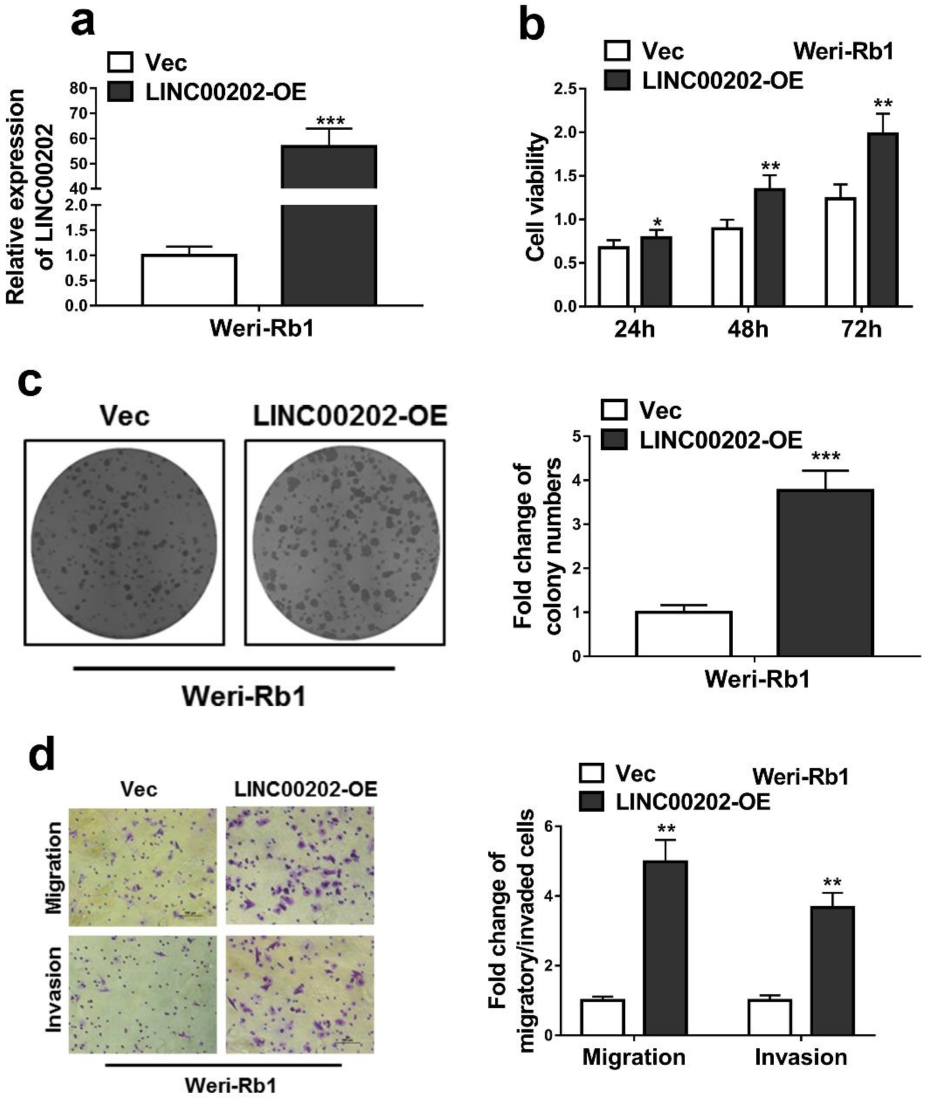

Figure S1. LINC00202 overexpression promotes RB cell proliferation, migration and invasion. Weri-Rb1 cells were transfected with LINC00202 overexpression plasmid (LINC00202-OE) or empty vector (Vec), followed by puromycin selection to establish the stable cell lines. The stable cells were then subjected to qRT-PCR analysis of LINC00202 expression (a), CCK-8 assay (b) and colony formation assay (c) to analyze cell viability, and transwell assays to examine cell abilities to migration and invasion (d). The data represent the mean ± SD from three independent experiments. ***P*<0.01; *** *P*<0.001.
