# Supplementary material for: Long Noncoding RNA LINC00202 Promotes Tumor Progression by Sponging miR-3619-5p in Retinoblastoma
Source: Cell Struct Funct. 2019 Mar 23;44(1):51–60. doi: 10.1247/csf.18033 (PMC11926405; doi:10.1247/csf.18033)
Supplement: Supplementary file 2 — Fig. S2 RIN1 is a crucial effector for RB progression. Weri-Rb1 and Y79 cells were transfected with RIN1 overexpression plasmid (RIN1-OE) or empty vector (Vec), and then subject to qRT-PCR analysis of RIN1 mRNA levels (a), Western blot analysis of RIN1 protein levels (b), CCK-8 assay (c, d) and colony formation assay (e) to analyze cell viability, and transwell assays to examine cell abilities to migration and invasion (f, g). Y79 cells were transfected with miR-3619-5p inhibitor combined with RIN1 siRNA, followed by qRT-PCR analysis of RIN1 mRNA levels (h), Western blot analysis of RIN1 protein levels (i), CCK-8 assay (j) and colony formation assay (k) to analyze cell viability, and transwell assays to examine cell abilities to migration and invasion (l). The data represent the mean±SD from three independent experiments. The data represent the mean±SD from three independent experiments. *P<0.05; **P<0.01; ***P<0.001. [file csf_44_18033_2.pdf]

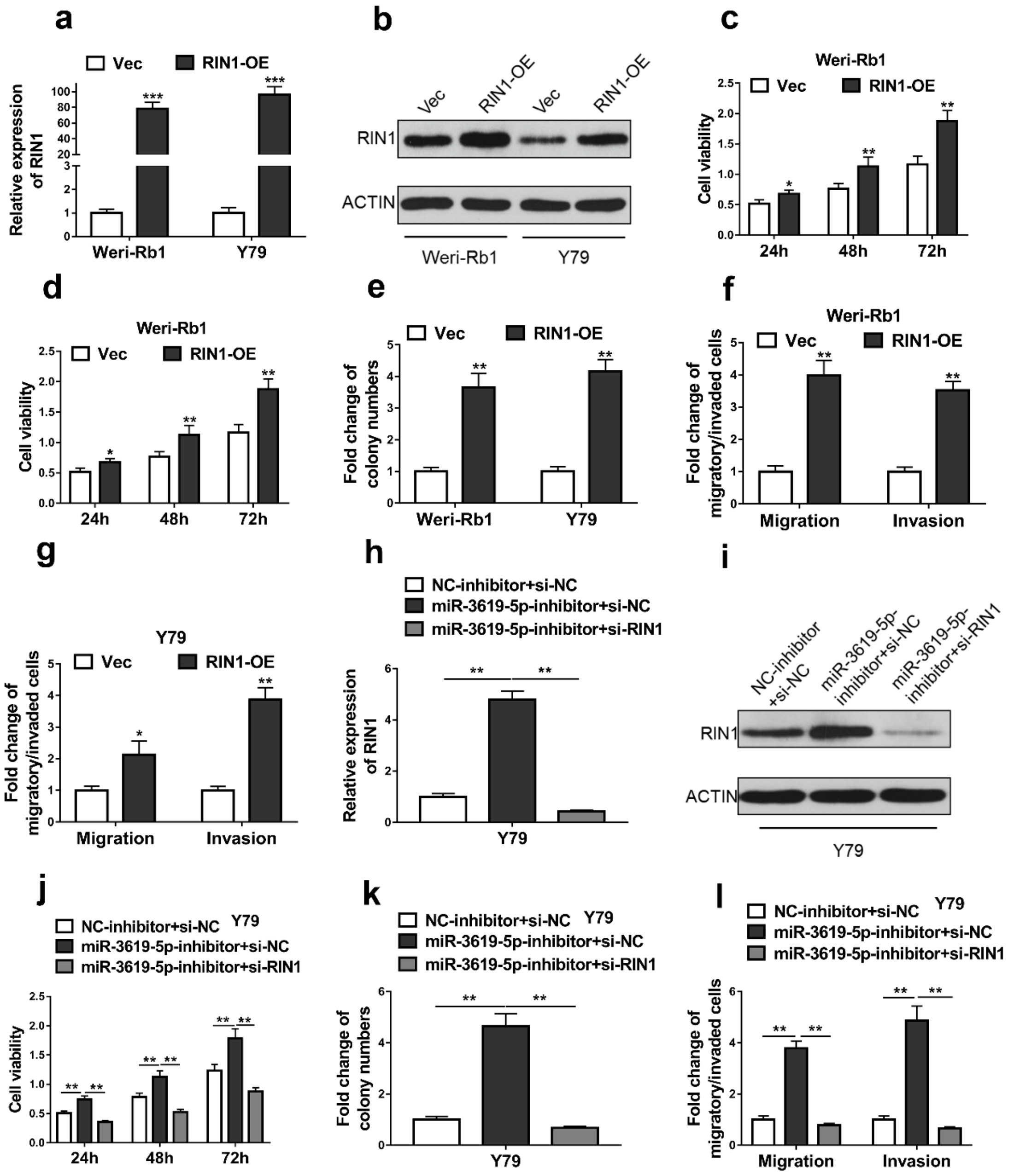

Figure S2. RIN1 is a crucial effector for RB progression.

Weri-Rb1 and Y79 cells were transfected with RIN1 overexpression plasmid (RIN1-OE) or empty vector (Vec), and then subject to qRT-PCR analysis of RIN1 mRNA levels (a), Western blot analysis of RIN1 protein levels (b), CCK-8 assay (c, d) and colony formation assay (e) to analyze cell viability, and transwell assays to examine cell abilities to migration and invasion (f, g). Y79 cells were transfected with miR-3619-5p inhibitor combined with RIN1 siRNA, followed by qRT-PCR analysis of RIN1 mRNA levels (h), Western blot analysis of RIN1 protein levels (i), CCK-8 assay (j) and colony formation assay (k) to analyze cell viability, and transwell assays to examine cell abilities to migration and invasion (l). The data represent the mean ± SD from three independent experiments. The data represent the mean ± SD from three independent experiments. **P*<0.05; ***P*<0.01; *** *P*<0.001.
